# Lifestyle modification and medication use among diabetes mellitus patients attending Jimma University Medical Center, Jimma zone, south west Ethiopia

**DOI:** 10.1038/s41598-023-32145-y

**Published:** 2023-03-27

**Authors:** Aster Wakjira Garedow, Tsiyon Mekoya Jemaneh, Addisalem Gebresilase Hailemariam, Gorfineh Teshome Tesfaye

**Affiliations:** 1 Jimma University, School of Pharmacy, Jimma, Ethiopia; 2 Jimma University, School of Nursing, Jimma, Ethiopia; 3 Jimma University, School of Medicine, Jimma, Ethiopia; 4 Jimma University Medical Center, Jimma, Ethiopia

**Keywords:** Diseases, Endocrinology, Health care, Medical research

## Abstract

Diabetes, a non-communicable metabolic disease, causes multiple complications and deaths worldwide. It is a complex, chronic disease that requires continuous medical care with multifactorial risk reduction strategies beyond glycemic control. Ongoing patient education and self-management support are critical for preventing acute complications and reducing the risk of long-term complications. There is ample evidence that healthy lifestyle choices, such as a healthy diet, moderate weight loss, and regular exercise, can maintain normal blood sugar levels and minimize diabetes-related complications. In addition, this lifestyle change has a major impact on controlling hyperglycemia and can help to maintain normal blood sugar levels. This study aimed to assess lifestyle modification and medication use in patients with diabetes mellitus at Jimma University Medical Center. Hospital-based prospective cross-sectional study was conducted from April 1 to September 30, 2021 among DM patients who have follow-up at diabetic clinic of Jimma University Medical Center. Consecutive sampling was used until the required sample size was achieved. Data were checked for completeness, then entered into Epidata version 4.2 software and exported to SPSS version 21.0. Pearson’s chi-square test was performed to determine the association between KAP and independent factors. Variables with a p value < 0.05 were considered significant. A total of 190 participants took part in this study with a response rate of 100%. In this study, 69 (36.3%) participants had good knowledge, 82 (43.2%) moderate knowledge and 39 (20.5%) poor knowledge, 153 (85.8%) had positive attitudes, 141 (74.2%) had good practice. Marital status, Occupational status and educational status were significantly associated with knowledge and attitude towards LSM and medication use. Marital status was the only variable that remained significantly associated with knowledge, attitude and practice towards LSM and medication use. The result of this study showed that more than 20% of the participants had poor knowledge, attitude, and practice towards medication use and LSM. Marital status was the only variable which remained to be significantly associated with KAP towards LSM and medication use.

## Introduction

Diabetes mellitus (DM) is a group of metabolic disorders characterized by hyperglycemia Diabetes mellitus (DM) is a group of metabolic disorders characterized by hyperglycemia resulting from defects in insulin secretion, action or both”. Diabetes mellitus is classified into two types namely; type 1 (T1DM) and type 2 diabetes mellitus (T2DM)^1^. Diabetes is a complex, chronic illness that requires continuous medical care with multifactorial risk-reduction strategies beyond glycemic control. Ongoing patient self-management education and support are critical for preventing acute complications and reducing the risk of long-term complications^2^.

The prevalence of DM is rapidly rising globally at a threatening rate. According to world health organization, 346 million individuals globally were diagnosed to have diabetes, among which 90% of individuals have T2DM. This value is expected to rise to 380 million individuals by the year 2025^3^, Globally; an estimated 422 million adults are living with DM, type 2DM makes up about 90–95% of all cases and the remaining 2–5% is type 1^4^. Throughout the last 20 years, the incidence of diabetes has been raised intensively in many parts of the world^5^. More than twelve million people were estimated to be living with diabetes in Africa which is projected to increase to 23.9 million by 2030^6,7^. The reasons for DM are urbanization, lack of physical activity, sedentary life style and obesity^8^. If not all but most of the factors are modifiable through proper lifestyle practices, drug taking behavior and screening for complications^9^.

In Ethiopia, the prevalence of diabetes was 3.5% in 2011, and the extrapolated prevalence in 2013 was 4.36%. It is also known that a large number of people remain undiagnosed, with an estimated number of undiagnosed cases reported to be 1.39 million people in 2013^6^ Inadequate knowledge about the disease, prognosis, complications and treatment results in poor glycemic control which in turn leads to increase in morbidity^10^. This increases the need for proper education regarding alterations in lifestyle (exercise and food), medication adherence, regular screening in patients with DM^11–13^.

The increase in the incidence of diabetes in developing countries follows the trend of urbanization and life style changes^14^. There was no attention given to diabetic education in Ethiopia, there were no diabetes nurse educators and diabetes dieticians in the country and those who provided health services for diabetes had no special training for diabetes care^15^. Control of the DM through a tight schedule of blood glucose and urine sugar monitoring, medication and adjustment to dietary condition need patient’s regular attention and discipline^16,17^. Proper metabolic conditions depend on several factors such as patient awareness on the pathophysiological aspect and those concerned with disease treatment, nutritional reduction, increased physical activity, regular foot inspection, signs and symptoms of hypoglycemia and prevention of chronic diseases, disease management in special situations and family support^18^. The present study assessed the knowledge, attitude and practice of DM patients regarding medication use and life style modification.

## Methods and participates

### Study setting and population

The study was conducted at Jimma University Medical Center (JUMC) from April 1 to September 30, 2021 among DM patients who have follow-up in the diabetic clinic of JUMC which is the only referral hospital, serves 20 million catchment areas. Patients were eligible for inclusion if they were greater than 18 of age and were willing to provide informed consent and excluded if they were unconscious and not willing to give consent. The dependent variable was KAP and independent variables included patients-related factors: age, sex, BMI, educational status, residence, monthly income, marital status and occupation.

### Study design, sample size determination and sampling technique

A hospital-based prospective cross sectional study was conducted. The sample size was calculated by using simple proportion formula and consecutive Sampling Technique was used until required sample size was obtained.

$${\text{n}} = \frac{{\left( {z \propto /2} \right)2p\left( {1 - p} \right)}}{d2}$$

where: n—required sample size, Z—standard deviation normal value at 95% CI which is 1.96, p—prevalence of KAP, p = 0.5, d—possible margin of error that can be tolerated 5% (0.05), q = 1 − p.

Thus; n = [1.96]^2^ * 0.5(0.5)/(0.05)^2^ = 384.

The source population of DM on follow up during 2020 in JUMC = N = 3052 which were < 10,000, we can use correction formula to calculate final sample size.

$${\text{n}}_{f} = \frac{n}{1 + n - 1/N}$$

n_f_ = =  384/1 + 384 − 1/3052 = 190

### Data collection procedures

Data were collected using a semi-structured questionnaire through interview and medical chart review. It includes socio-demographic, medication-related, and laboratory as well as clinical and diseases-related characteristics of the patient. Data was collected by four data collectors by following DM patients monthly and supervised after training was given on the detail of data collection tools/checklists for 2 days before data collection started. For KAP analysis towards DM the questionnaires have four major parts: socio-demographic information part, Health Seeking Behaviour, Health Status/occupational characteristics part and three domains (Knowledge, Attitude and practice). The knowledge aimed to assess causes, identification and management of DM. The item in the domain had two or four options (Yes, No and/or ‘Do Not Know’ and ‘Unsure’)). To assess the attitude and practice the questionnaires, we used items with yes or no options or Strongly agree, Agree, Not sure, Disagree, Strongly disagree .

### Data quality control and assurance

The International Council on Harmonization Guidelines for Good Clinical Practice and the Declaration of Helsinki's guiding principles will both be followed during the study's execution. The following specific ethical concerns were taken into account during the procedure. These include the fact that participation is voluntary, getting written agreement, after a necessary description of the goal, benefit, and risk of the study, as well as the subject's right to decide whether or not to participate. preserving privacy by omitting participant names in favor of a code. An independent party developed the surveys in English, had them translated into Amharic and the native language Afan Oromo, and had those translations returned to English. In ensure uniformity, the surveys were written in English, translated into Amharic and the regional language Afan Oromo, and then back into English. Prior to the actual data collection, a pre-test was carried out on 5% of the study participants by randomly chosen patients to assess the validity and consistency of the structured data collection format. Data were compiled, cleaned up, programmed, and consistency-checked. The supervisor attentively observed each step of data collecting and recording, and any gaps were immediately shared with the data collectors.

### Data processing and statistical analysis

Data was checked for completeness, coded, cleaned compiled then entered to Epidata version 4.2.0.0 software and exported to SPSS version 21.0 for analysis. Descriptive statistics were computed and presented in text and tables. Pearson chi-square was done to see the association between KAP and independent factors. Variables with a p value < 0.05 were considered as significant.

### Ethical consideration

Institutional review board approval was obtained from Jimma University and written informed consent was obtained from each study participant and guardians of study participants who were illiterate or uneducated.

### Definition of terms

Knowledge: understanding of subjects related to diabetes or level of information towards diabetes mellitus.

Good knowledge: Those who scored above 15 knowledge questions [^19,20^] [30].

Moderate knowledge: Those who scored 11–14 knowledge questions^20^

Poor knowledge: Those who scored 0–10 knowledge questions^20,21^

Attitude: The way of Diabetes Mellitus patients feels about the disease and its management.

Positive attitude: Score of 18-25attitudes point^19^

Negative attitude: Score of 0–17 attitude point^22^

Practice: The habit which the diabetic patients exhibits

Good practice: Score of 5–9 practice point^22^

Poor practice: Score of 0–4 practice point^17^

Life style modification: Refers to the change in living pattern of diabetes to reduce complication of disease and for better outcome of their medication, include non-pharmacological management such as diet modification, and exercise design to treat problem of DM patients^23^.

## Result

### Sociodemographic characteristics of the study participants

During 6 month study period, 190 DM patients were included. Most of study participants were in age group of 41–50 years with mean age of 45.83 ± 17.7. Majority of the study participants 115 (60.5%) were males, 65 (63.5%) had no regular income, 108 (56.8%) were living in rural area and 125 (65.8.%) were married. Most of study participants were 53 (51.5%) farmers and 73 (38.4%) had primary educations (Table 1).

**Table 1:** Distribution of study participants by socio-demographic characteristic at chronic follow up at JMC Jan 3–20 2021.

Variable		Number (N) = 190	Percent (%)
Sex	Male	115	60.5
	Female	75	39.5
Residence	Rural	108	56.8
	Urban	82	43.2
Age (in year)	18–30	28	14.7
	31–40	36	18.9
	41–50	51	26.8
	51–60	38	20.0
	> 60	37	19.5
Religion	Catholic	19	9.1
	Muslim	83	39.7
	Orthodox	64	30.8
	Protestant	43	20.6
Occupation	Government employee	27	14.2
	Farmer	46	24.2
	Student	28	14.7
	Merchant	30	15.8
	House wife	59	31.1
Marital status	Single	32	16.8
	Married	125	65.8
	Divorced	11	5.8
	Widowed	22	11.6
Educational status	No formal education	58	30.5
	Primary	73	38.4
	Secondary	24	12.6
	College and above	35	18.4
Monthly in come in birr	< 1000	80	41.8
	1001–1999	35	18.4
	> 3000	75	39.5

### Clinical characteristics of respondents

According to background information obtained from patient profile 58 (30.5)% had Type-I DM and 132 (69.5% ) had Type-II DM. Duration of disease since diagnosed 0–4 years 93 (41%), 5–9 years 77 (40.5%), 10–14 years 24 (12.6% ) and above 15 years 11 (5.8)% (Table 2).

**Table 2:** Distribution of diabetics by type of DM, and duration since diagnosis from Health background at JMC Jan 3–20-/2021.

Variables		Male (N)	Female (N)	Total (N)
Type of DM	I	32	26	58
	II	83	49	132
	Total	115	75	190
Duration of DM in years	0–4	45	33	78
	5–9	43	34	77
	10–14	19	5	24
	> 15	8	3	11
	Total	115	75	190

### Knowledge regarding diabetes and management

Regarding knowledge of respondent about Hyperglycemic symptoms, 149 knows that hyperglycemia causes increased thirst, 57 about increased urination, 51 about loss of consciousness, and 11 increased appetites, 35 about loss of weight and about 9 of them don’t know the symptoms at all. Concerning symptoms of hypoglycemia 66 know that sweating is caused by hypoglycemia, 76 chills/rigors, 110 loss of consciousness 73 palpitation and 7 don’t know any symptom (Table 3).

**Table 3:** Distribution of diabetics towards the knowledge of hypoglycemia and hyperglycemia at JMC DM clinic from Jan 3–20 2021.

Symptom of hypoglycemia		Male (N)	Female (N)	Total (N)	Symptom of hyperglycemia		Male (N)	Female (N)	Total (N)
Sweating	Yes	45	31	66	Increased thirst	Yes	89	60	150
	No	70	44	114		No	26	14	40
Palpitation	Yes	46	27	73	Increased urination	Yes	80	50	130
	No	69	48	117		No	35	25	60
Loss of consciousness	Yes	69	41	110	Loss of consciousness	Yes	21	17	38
	No	46	34	80		No	94	58	152
Chills (rigors)	Yes	46	30	76	Loss of weight	Yes	24	11	35
	No	69	45	115		No	91	64	155
Don’t know		3	4	7	Increased appetite	Yes	42	22	64
						No	73	53	126
					Don’t know		4	5	9

### Knowledge regarding life style modification and management

Concerning alcohol intake 77.4% know that it’s important to reduce alcohol intake, 75.3% know that regular exercise were important, 42.1% knows importance of smoking cessation, 65.5% importance of alcohol cessation and 52.6% about importance of diet control, 69.09% of study participants know that DM patient should avoid sugar and other sweet diets but 30.91% know it is important to use it. Concerning exercise 60.91% have negative response, the rest 39.09% of them have positive response (Table 4).

**Table 4:** Knowledge of DM patients on life style modification, at JMC DM clinic from Jan 3–20 2021.

		Male N (%)	Female N (%)	Total (%)
Alcohol intake	Yes	85 (44.7%)	62 (32.6%)	147 (77.4%)
	No	30 (15.7%)	13 (10%)	43 (22.6%)
Lifestyle modification				
Weight reduction (regular exercise)	Yes	87 (45.8%)	56 (29.5%)	143 (75.3%)
	No	28 (14.7%)	19 (10%)	47 (24.7%)
Smoking cessation	Yes	53 (27.9%)	27 (14.2%)	80 (42.1%)
	No	62 (32.6%)	48 (25.3%)	110 (57.9%)
Cessation alcohol intake	Yes	75 (39.5%)	50 (26.3%)	125 (65.8%)
	No	40 (21.1%)	25 (13.2%)	65 (34.2%)
Diet control	Yes	61 (32.1%)	39 (20.5%)	100 (52.6%)
	No	54 (28.4%)	36 (18.9%)	90 (47.3%)
	Don’t know	11 (5.8%)	9 (4.7%)	4 (10.5%)
Is it important to use sugar and sweet diets	Yes	37 (19.5%)	26 (13.6%)	63 (33.1%)
	No	72 (37.9%)	45 (23.6%)	117 (61.5%)
	I don’t know	6 (3.1%)	4 (2.1%)	10 (5.2%)
Doing exercise	Yes	78 (22.73%)	47 (16.36%)	125 (65.8%)
	No	19 (11.2%)	18 (6.37%)	37 (19.4%)
	Don’t know	18 (27.27%)	10 (16.07%)	28 (14.8%)

### Knowledge regarding medication, side effects and management

Regarding knowledge of diabetes on their type of medications 40 respondents uses insulin with oral hypoglycemic agents, 86 use oral hypoglycemic agents, 57 use insulin only and 7 uses other**.** Concerning knowledge of study participants on medication side effects 27.4% know that medications of diabetes mellitus can cause hypoglycemia, 12.1% allergic reaction, 18.4% anemia and 16.3% others (GI upset, headache and nausea). Regarding the way in which they manage the side effects, 43.7% manage by visiting hospital, 21.6% by taking another medications, 18.9% stop taking medication, 15.8% by other ways (changing eating style and taking rest) (Table 5).

**Table 5:** Knowledge of patients on medication side effects and management at JMC, DM clinic from Jan 3–20-/2021.

	Male N (%)	Female N (%)	Total (%)
Observed side effects			
Allergic reaction	13 (6.8%)	10 (7.7%)	23 (12.1%)
Anemia	15 (7.9%)	20 (10.5%)	35 (18.4%)
Hypoglycemia	29 (15.2%)	23 (12.2%)	52 (27.4%)
Weight loss	34 (17.9%)	15 (7.9%)	49 (25.8%)
Others weight (gain, GIT upset)	24 (12.6%)	7 (3.7%)	31 (16.3%)
Total	115 (61.2%)	75 (38.8%)	190 (100%)
Management of side effects			
Go to hospital	46 (24.2%)	37 (19.5%)	83 (43.7%)
Taking another medication	25 (13.2%)	16 (8.4%)	41 (21.6%)
Stop medication	20 (10.5%)	16 (8.4%)	36 (18.9%)
Others (change eating style and taking a rest)	24 (12.6%)	6 (3.2%)	30 (15.8%)

### Attitude and practice of respondents

Regarding the attitude of patients, 142 (74.7%) of them had positive attitude on life style modification, 185 (97.4%) of them had positive attitude on regular visit of DM clinic, 156 (82.1%) had positive attitude on diabetes care and treatment, 95.78% had positive attitude on lifelong treatment of their DM, 51.1% of respondents had positive attitude on continues use of medication; even if their glucose is controlled (Table 6).

**Table 6:** Self- care attitude of diabetes patient to control blood glucose and disease complication at JMC, DM clinic from Jan 3–20-/2021.

Assessment of attitude perception	SA	A	N	D	SD
Does DM can be treated	6	150	21	12	1
DM requires long term treatment	19	163	6	2	0
Could you stop your medication whenever your blood glucose is controlled	3	31	59	97	0
Could life style modification such as dietary modifications, moderation of alcohol intake and cigarette smoking cessation Play role in treatment of DM	3	139	44	4	0
Regular visit of clinic has benefit for DM patients	50	135	5	0	0

Regarding practice of study participants 152 (80%) participants have positive response for storage and use of medication, and 172 (90.5%) for regular blood taste. Majority have positive response to avoid alcohol drinking, stop smoking, inspection of foot daily for any color Change or any wound and go to clinic as appointed (Table 7).

**Table 7:** Self- care practice of diabetes patient to control blood glucose and disease complication at JMC, DM clinic from Jan 3–20-/2021.

Variable		Male	Female	Total
Regular exercise	Yes	69	43	112
	No	46	32	78
Drinker	Yes	31	11	42
	No	84	64	148
Diet control	Yes	75	47	122
	No	40	28	68
	Yes	23	11	34
	No	77	76	153
Clinic visit	Yes	105	67	172
	No	10	8	18
Regular urine test	Yes	67	38	105
	No	48	37	85
Regular blood test	Yes	106	66	172
	No	9	9	18
Inspection of foot	Yes	59	42	101
	No	56	33	89
Medication use and storage	Yes	96	56	152
	No	19	19	38

### Association between KAP and dependent variables

Pearson chi-square association shows that occupation (p = 0.014), marital status (p =  < 0.001) and educational status (p = 0.013) were significantly associated with knowledge towards LSM and medication use (Table 8).

**Table 8:** Association of knowledge with socio demographic variables towards LSM and medication use among DM patients at JMC, January 3–20-2021.

Variables		Status of knowledge			p value
		Good knowledge	Moderate knowledge	Poor knowledge	
Sex	Male	43	50	22	0.829
	Female	26	32	17	
Age	18–30	13	13	2	0.092
	31–40	13	14	9	
	41–50	23	21	7	
	51–60	14	15	9	
	> 60	6	19	12	
Religion	Muslim	14	45	25	0.26
	Orthodox	15	30	9	
	Protestant	8	4	4	
	Catholic	5	3	1	
Occupation	Government employee	9	15	3	0.014
	Merchant	11	15	4	
	Farmer	17	14	15	
	Student	17	9	2	
	House wife	15	29	15	
Marital status	Married	45	55	25	< 0.001
	Single	20	11	1	
	Divorce	3	5	3	
	Widowed	1	11	10	
Educational status	No formal education	13	25	20	0.013
	Primary	31	28	14	
	Secondary	12	10	2	
	College and above	13	19	3	
Residence	Urban area	31	34	17	0.911
	Rural area	38	48	22	
Monthly income	< 1000	29	30	21	0.400
	1001–2999	14	17	4	
	> 3000	26	35	14	

### Factors associated with attitudes

Pearson chi-square association shows, age with p value of (p =  < 0.001), occupational status (p = 0.04), marital status (p =  < 0.001), and educational status (p =  < 0.001) were significantly associated with the attitude of participants towards LSM and medication use of diabetes patient (Table 9).

**Table 9:** Pearson chi-square association of attitude with socio demographic variables towards LSM and medication use among DM patients at JMC, January 3–20-2021.

Variables		Status of attitude		p value
		Positive attitude	Negative attitude	
Sex	Male	96	19	0.259
	Female	97	6	
Age	18–30	27	1	< 0.001
	31–40	34	2	
	41–50	31	3	
	51–60	23	74	
	> 60	12	15	
Religion	Muslim	91	20	0.269
	Orthodox	49	5	
	Protestant	14	2	
	Catholic	3	0	
Occupation	Government employee	27	0	0.04
	Merchant	24	6	
	Farmer	33	13	
	Student	27	1	
	House wife	52	7	
Marital status	Married	113	12	< 0.001
	Single	31	1	
	Divorce	7	4	
	Widowed	12	10	
Educational status	No formal education	38	20	< 0.001
	Primary	68	5	
	Secondary	23	1	
	College and above	34	1	
Residence	Urban area	75	7	0.051
	Rural area	88	20	
Monthly income	< 1000	69	11	0.071
	1001–2999	26	9	
	> 3000	68	7	

### Factors associated with practices

Pearson chi-square association shows, age with p value of (p = 0.03), and marital Status (p = 0.049) of the participants were significantly associated with practice toward LSM and medication use of diabetes study participants (Table 10).

**Table 10:** Pearson chi-square association of practice with socio demographic variables towards LSM and medication use among DM patient at JMC, January 3–20 2021.

Variables		Status of practice		p value
		Good practice	Poor practice	
Sex	Male	90	25	0.114
	Female	51	24	
Age	18–30	24	4	0.03
	31–40	32	4	
	41–50	36	15	
	51–60	27	11	
	> 60	22	15	
Religion	Muslim	81	30	0.132
	Orthodox	37	7	
	Protestant	14	3	
	Catholic	9	0	
Occupation	Government employee	23	4	0.08
	Merchant	23	7	
	Farmer	30	16	
	Student	25	3	
	House wife	40	19	
Marital status	Married	95	30	0.049
	Single	28	4	
	Divorce	8	3	
	Widowed	10	12	
Educational status	No formal education	38	20	0.108
	Primary school	54	19	
	Secondary school	18	6	
	College and above	31	4	
Residence	Urban area	62	20	0.701
	Rural area	79	29	
Monthly income	< 1000	57	53	0.723
	1001–2999	27	8	
	> 3000	57	18	

## Discussion

Majority of respondents in this study came from the age groups 41–50 years (26.8%). Which is similar with study conducted at Adama hospital^21^. This is reflective of the fact that the etiology of diabetes mellitus usually at old age^24,25–27^. In this study respondents with no formal education consists 58 (30.5%) and only (18.4%) respondents with college and above education. This result may be the direct consequence of scarcity of higher education system in Ethiopia in the past^23^. This shows that there is some improvement in educational status of DM patient compared with the study conducted in 2011 at JUSH which reported as there was no attention given to diabetic education in Ethiopia^22^. Majority of respondents in this study had income less than 1000 birr 80 (42.1%). This low income among respondents could limit their accessibility and affordability of a well-balanced diet and healthy food and it was considered as the main factors (barrier) to their practice of life style modification and proper use of their medications. This finding was in keeping with study conducted in Gondar; in which majority of the study participants 139 (43%) had very low monthly income^28^.

In terms of Knowledge Assessment, 69 (36.3%) had good knowledge, 82 (43.2%) moderate knowledge and 39 (20.5%) poor knowledge of LSM and medication use of the DM patient. In contrast to this finding study done in Western Nepal shows that knowledge, attitude and practice among diabetes patients were low^15^. This discrepancy might be due to study setting, study design, and geographical area .Regarding Knowledge toward life style modification, 75.3% respond that regular exercise where important for DM patient, and 42.1% knows importance of smoking cessation, 65.5% cessation of alcohol importance and 52.6% about importance of diet control. In line with this Study done in Malaysia identified a good KAP score of life style modification required for diabetes patients^10^. Regarding the attitude of respondent, 142 (74.7%) of them have positive attitude on life style modification, 185 (97.4%) of them had positive attitude on regular visit of DM clinic, 156 (82.1%) had positive attitude on diabetes care and treatment, 95.78% lifelong treatment of DM, 51.1% of respondents have positive attitude on continues use of medication; even if their glucose is controlled. This finding is similar to study done in South Africa in which the majority of respondents 92.7% and 51.6% had positive attitude on lifestyle modification and medication use respectively^28^.

Among study respondent about 141 (74.2%) had good practice of LSM and medication use, while 49 (24.8%) had poor practices which is comparative with study done in Gondar, Ethiopia, that were 74.4% showed good practice^21^. However, it was higher than the study done in Harar, Ethiopia, in which 39.2% had good self-care practice [30]. This might be due to difference in socio demographic, study design, study setting and access to health education programs. In this study, occupation status and educational status showed a significant association with knowledge, whereas age, occupation status, marital status and educational status showed a significant association with attitude, age and marital Status showed a significant association with practice towards LSM and medication use. This finding was similar to the studies conducted in different settings^15,20,22^.The difference among different study findings may be difference literacy of patients, training given to the patients, availability of information on different facilities.

## Limitations of study

The current study is only focused on patients aged 18 years and above, conducted at single setting, JMC and it did not consider DM patients who did not visit the health institutions during the study period.

## Conclusion

The result of this study showed that more than 20% of the participants had poor knowledge, attitude, and practice towards medication use and LSM. Marital status was the only variable which remained to be significantly associated with all three: knowledge, attitude and practice towards LSM and medication use.

## Recommendation

Lifestyle modification has a great role in the prevention and control of blood glucose raised. But, there has to be much to be done on this, since its prevalence is increasing rapidly in worldwide especially in developing nations on the top of changed lifestyle, the deficit in the knowledge, attitude, and practice towards lifestyle modification. On the basis of the study findings, the following recommendations were forwarded Health education about diabetes and types of lifestyle modification has to be strengthened. Better to create awareness to the community through health education about the importance of physical exercise, weight loss, feet care, healthy dietary habit and cessation of smoking.

## Data Availability

Readers who will require data and materials of the current study can communicate and get from the corresponding author with a reasonable request.

## Abbreviations

DM: Diabetes mellitus
FBS: Fasting blood sugar
IDDM: Insulin dependent diabetes mellitus
IDF: International diabetic federation
JUMC: Jimma University Medical Center
KAP: Knowledge, attitude and practice
LSM: Life style modification
NIDDM: Non-insulin dependent diabetes mellitus
PI: Principal investigator
SPSS: Statistical package for social science
